# Prevalence of Pressure Injuries Nationwide from 2009 to 2015: Results from the National Inpatient Sample Database in Korea

**DOI:** 10.3390/ijerph16050704

**Published:** 2019-02-27

**Authors:** Gyeong Hoe Kim, Jin Yong Lee, Jayeun Kim, Hyun Joo Kim, Ji-Ung Park

**Affiliations:** 1 Department of Plastic and Reconstructive Surgery, Seoul National University Hospital, Seoul 03080, Korea; hwe0216@gmail.com; 2 Public Health Medical Service, Seoul Metropolitan Government-Seoul National University Boramae Medical Center, Seoul National University College of Medicine, Seoul 07061, Korea; jylee00@snu.ac.kr; 3 Institute of Health Policy and Management, Medical Research Center, Seoul National University, Seoul 03080, Korea; 4 Institute of Health and Environment, Seoul National University, Seoul 08826, Korea; kimjayeun@gmail.com; 5 Department of Nursing Science, Shinsung University, Dangjin 31801, Korea; hyjkim2012@gmail.com; 6 Department of Plastic and Reconstructive Surgery, Seoul Metropolitan Government-Seoul National University Boramae Medical Center, Seoul 07061, Korea

**Keywords:** pressure injury, trend, prevalence, socioeconomic status, aging, healthcare system

## Abstract

This study aimed to investigate the prevalence pattern of pressure injuries (PIs), or ‘sores’, in South Korea and investigate the factors affecting its development. We estimated the annual prevalence of PIs using the National Inpatient Sample (NIS) data extracted from the Health Insurance Review and Assessment Service (HIRA) database from 2009 to 2015. Multivariable logistic regression was performed to examine the association between hospitalization and socio-demographic characteristics, such as sex, age, type of health insurance, Charlson Comorbidity Index (CCI), and plegia comorbidity. We found that inpatients with PIs make up to 0.86% of the total population in South Korea in 2015, which had shown a steady increase from the previous years. And male, old age, low socioeconomic status (SES), and the patients’ severity such as high CCI and the plegia comorbidity were strongly associated with hospitalization due to PI. Based on our results, it would be anticipated that the medical cost for treatment and management of PIs will increase in the future, and it will be accelerated due to the rapidly aging society. In addition, patients in low SES and patients with severe comorbidities would be relatively more burdensome, threatening their household economy and further reducing the quality of life. Therefore, PIs should not be overlooked as the responsibility of just the nursing care professionals but should be recognized as one of the serious societal problems. The establishment of an intense medical care system is needed not only to reduce the prevalence of PIs but also to increase the awareness in people with PI patients.

## 1. Introduction

A pressure injury (PI), also referred to by the public as a “sore”, is a localized injury to the underlying tissue, usually over a bony prominence, due to pressure. As well as ischemic damage due to the compression of the artery, the occlusion of lymphatic vessels, shearing or friction force, and unregulated moisture are known to cause a PI [1,2]. The elderly, acutely ill, and spinal cord–injured patients are known to be vulnerable to PIs [3]. A PI can lead to chronic pain, as well as premature death if accompanied by osteomyelitis or sepsis. Prevention and treatment of PIs require labor-intensive care by caregivers such as frequent position changes, nutritional support, and incontinence management [4]. Furthermore, because the contributing factors such as immobility cannot be cured, PI recurs and repeatedly worsens, which eventually results in a rise in national healthcare costs. In the United States, medical costs for management of PIs range from $500 to $70,000 per patient, totaling an estimated $11 billion annually [5]. To monitor the occurrence of PIs and to assess the quality of hospital nursing, there have been prevalence studies of various types in several countries. Although the prevalence of PIs reported in these studies ranged from 1.58% to 18.1%, they were for a specific group of inpatients rather than the total population in a country [6,7,8,9,10,11,12]. Moreover, most studies were conducted in hospitals that voluntarily participated in the study, and not randomly selected hospitals. Similarly, in South Korea, there has been a prevalence study of patients in the emergency rooms or intensive care units, but the nationwide prevalence of PIs has not yet been investigated [13]. One of the things to consider when establishing a care system associated with PIs is how to allocate the limited medical resources. In terms of distribution of medical resources, the socio-economic status (SES) of a patient must be considered on the basis of the principle of social security. In South Korea, all citizens are covered by the mandatory health insurance system from birth to death. Based on their SES, most people are designated as national health insurance (NHI) beneficiaries, while those from a lower socioeconomic background who are recipients of the National Basic Livelihood Security System in South Korea are designated as medical aid (MA) beneficiaries. If PIs are associated with individuals belonging to the low SES as seen with hypertension or diabetes [14,15,16,17], it suggests the need for developing complementary health policies to ameliorate the socioeconomic inequalities.

For these reasons, it is needed to investigate the prevalence of PIs in South Korea’s total population based on the diagnostic information registered at the Korean Health Insurance Review and Assessment Service (HIRA) (http://www.hira.or.kr). It included demographic studies including the sex and age of the patient. Second, the prevalence of PIs with regards to the region and type of medical institutions based on the type of health insurance was examined. In addition, we also investigated the various comorbidities associated with PIs on the basis of concurrently registered diagnoses. Finally, we examined the factors affecting the development of PI through logistic regression analysis. In summary, this study aimed to examine the prevalence pattern of PIs from 2009 to 2015 in South Korea and investigate the factors affecting its development.

## 2. Materials and Methods

### 2.1. Data Source

We used the data from the National Inpatient Sample (NIS) database, the official health insurance claim data published annually by HIRA. For the purpose of this study, we focused on the study period between 2009 and 2015. The database contains a representative sample of the HIRA claims data, including 13% of annual inpatient claims (about 700 thousand inpatients) and 1% of annual outpatient claims (about 400 thousand outpatients) made in Korea [18,19].

### 2.2. Operational Definition of Study Population

Inpatients with PIs were defined using the diagnosis code L89, based on the International Classification of Diseases-10th (ICD-10th) Revision. In the process of defining PI inpatients, we used not only the primary or subsidiary codes but included all the diagnostic information provided by the separate forms in the medical service claims data. The total number of inpatients was calculated from 2009 through 2015, based on age group, sex, type of health insurance, level and regional location of the medical institution, and comorbidities. In the analyses, patient age was divided into 4 groups: 0–39, 40–64, 65–74, and 75+. Types of health insurance were divided into NHI and MA. About 4% of South Korea’s population are covered by the MA program and receive additional public assistance, while the remaining 96% are covered by the NHI program [20]. While the level of medical institutions where the inpatients were treated at was categorized into a tertiary teaching hospital, general hospital, hospital, and clinic (In South Korea, medical institutions with less than 30 beds are also classified as clinic in Korea), the regional location of the medical institution was either metropolitan or non-metropolitan. 

The Charlson Comorbidity Index (CCI), which predicts the one-year mortality for a patient who may have a range of comorbid conditions, such as heart disease, AIDS, or cancer [21], was determined for each subject based on the medical care utilization records for the study period 2009–2015. The NIS database we used, is a sampling dataset extracted from the total Korean population. The data comprises 1% of outpatient and 13% of inpatient claims, and sampling extraction is conducted annually using patient unit stratification, according to sex and age in five-year increments. Due to annual basis sampling extraction, the history of individual’s comorbidities for several consecutive years will be summed up in CCI calculation. Therefore, in the process of calculating CCI, we applied a cross-sectional approach due to the relatively short study period to check the history of medical service utilization for each patient. If a patient used medical services for diseases that CCI accounts for throughout each year, those were considered as comorbidities without taking into account the temporal context. In addition to CCI, several diseases which were clinically related to PIs were reviewed using the ICD-10th codes for quadriplegia (G825), hemiplegia (G819), monoplegia (G831, G832, G833), paraplegia (G822), flaccid paralysis (G810, G820, G823), spastic paralysis (G811, G821, G824), diabetes (E10, E11), and sepsis (A40, A41, R572, R650, R651).

### 2.3. Statistical Analysis

Frequency analyses were performed to evaluate the distribution and annual standardized number (per 100,000 individuals) of PI inpatients from 2009 to 2015 for the overall population. Standardized number of annual PI inpatients was calculated by applying the age and sex structure of 2015 census data surveyed by Statistics Korea. The annual trends of the standardized number of inpatients with PI between 2009 and 2015 were analyzed using simple linear regression. Multivariable logistic regression was performed to examine the association between hospitalization and socio-demographic characteristics, such as sex, age, type of health insurance, CCI, and plegia comorbidity. In order to acquire a consistent association between the diagnosis of PI and socio-demographic characteristics, we conducted a regression analysis biennially in 2009, 2011, 2013, and 2015. All analyses were completed using the SAS, version 9.4 (SAS Institute, Inc., Cary, NC, USA). All statistical tests were 2-sided, and a *p* value of < 0.05 was considered statistically significant.

### 2.4. Ethical Statement

This study was exempted from approval by the Institutional Review Board of Seoul National University Boramae Medical Center (No. 07-2018-1). Informed consent was not obtained because patient records/information was anonymized and de-identified prior to analysis.

## 3. Results

### 3.1. Number of Inpatients with PI and Its Standardized Prevalence from 2009 to 2015

Total study populations and number of PI inpatients from 2009 to 2015 in South Korea are presented in Table 1. The total study population has steadily increased from 1,116,040 in 2009 to 1,293,144 in 2015, except for the period from 2012 to 2013, which was marked by almost no growth in the Korean population. The prevalence of PI inpatients among total population steadily increased from 0.74% in 2009 to 0.86% in 2015, except for the period from 2010 to 2012. The standardized number of 861 from the data in 2015 means that 124,629 individuals of the total population in South Korea were diagnosed with PIs when sampling weight was applied.

**Figure 1:**
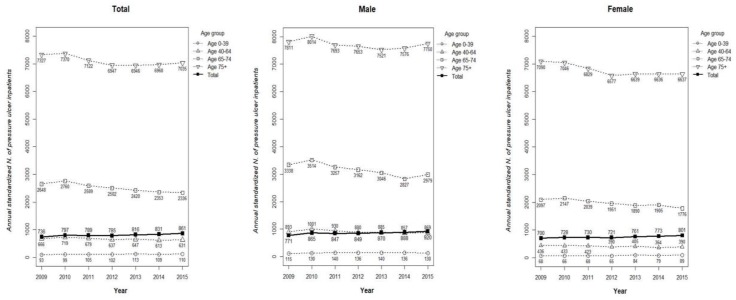
Standardized number of PI inpatients per 10^5^ populations by age and sex using the population data from the Statistics Korea.

The standardized numbers of PI inpatients per 10^5^ populations by age and sex using population data from the Statistics Korea are reported in Figure 1. The annual standardized number of PI inpatients showed a significant difference when classified into four groups according to age. In 2015, the standardized number of PI inpatients older than 75 was the 7035, which was the highest among all age groups, followed by 2336 for the 65–74 age group, 631 for the 40 to 64 age group and 110 for patients younger than 39 years. This comparative trend was the same for all the years evaluated. When classified by sex, the standardized number of male inpatients with PIs tended to be higher in comparison to female inpatients. 

### 3.2. Number of PI Inpatients by SES Including the Type of Health Insurance, Regional Location, and Level of Medical Institution

The numbers of PI inpatients by the type of health insurance, regional location and level of medical institution are reported in Table 2. When classified by the type of health insurance, 22% of the PI inpatients were MA beneficiaries in 2015, which was less than the 28.2% in 2009. This is a remarkably high proportion considering that only 4% of the total populations are MA beneficiaries [20].

While 46.4% of the PI inpatients visited metropolitan hospitals in 2015, the remaining visited non-metropolitan hospitals. When the group was classified based on the type of health insurance, 46.9% of the NHI beneficiaries visited metropolitan hospitals in 2015, which was higher than 44.4% MA beneficiaries who did the same. This comparative trend was the same for all the years evaluated.

In 2015, 19.6%, 31.1%, 43.3% and 6.0% of the PI inpatients visited a tertiary teaching hospital, general hospital, hospital, and clinic, respectively. When the group was classified based on the type of health insurance, there was a difference in the distribution of medical institution grades visited by the patients. In 2015, 12.0% of MA beneficiaries visited a tertiary teaching hospital, which was lower than 21.3% of NHI beneficiaries who did the same. Among the PI inpatients who were MA beneficiaries, 32.1 % and 50.0% used a general hospital and hospital, respectively, which was higher than 30.9% and 41.8%, respectively used by the NHI beneficiaries. This comparative trend was seen for all the years evaluated.

### 3.3. Average CCI of PI Inpatients and the Number of PI Inpatients with Special Comorbidities

The average CCI of PI inpatients and the number of PI inpatients with special comorbidities are reported in Table 3. The average annual CCI of PI inpatients was between 2.0 and 2.3. The proportion of patients with plegia among the total PI inpatients was 13.8% and had steadily dropped from 17.4% in 2009. When classified by the type of plegia, the proportion of patients with hemiplegia was highest at 5.8%, followed by 3.9% with quadriplegia, 1.5% with paraplegia, 0.4% with monoplegia. This comparative trend was the same throughout the years. The proportion of patients with spastic paralysis was 2 to 3 times higher than that of patients with flaccid paralysis in all years. The proportion of patients with diabetes among the total PI inpatients was 12.7% in 2015, which was higher than the reported 10.3% in 2009. The proportion of patients with sepsis among the total PI inpatients was 12.1% in 2015, which was slightly higher than the 11.9% observed in 2009.

### 3.4. Logistic Regression Analysis between PI Diagnosis and Socio-Demographic Factors among the Overall Population that Used Medical Services between 2009 and 2015 in Korea

The results of logistic regression analysis between the diagnosis of PI and socio-demographic factors are reported in Table 4. In 2015, the odds ratio between PI diagnosis and female sex was 0.85 (0.83–0.86) compared to the male sex. The odds ratio between PI diagnosis and age groups 40 to 64, 65 to 74, and over 75 were 0.65 (0.63–0.67), 1.78 (1.72–1.84), and 5.29 (5.13–5.46), respectively, when compared to that of the group under 39 years of age. The odds ratio between PI diagnosis and MA beneficiaries was 1.46 (1.43–1.49) compared to that of NHI beneficiaries. The odds ratio between PI diagnosis and a CCI of 1 to 2, and over 3 was 1.10 (1.07–1.12), and 1.99 (1.94–2.04), respectively, when compared to a CCI of 0. Finally, the odds ratio between PI diagnosis and plegia comorbidity was 1.88 (1.81–1.95). In summary, the odds ratio which indicates the degree of association for PI diagnosis was over 1 when the patient was aged >65 years, an MA beneficiary, had a high CCI, and with plegia comorbidity. As the age and CCI of patients increased, the odds ratio also became bigger. The odds ratio was under 1 in a female patient.

## 4. Discussion

In this study, we found that the inpatients with PIs make up to 0.86% of the total population in South Korea in 2015, which had shown a steady increase from the previous years. Old age and low SES as determined by the type of health insurance were highly associated with a high prevalence of PIs. We also found that the higher the CCI was, the more relevance it had with the diagnosis of PI, especially with the plegia comorbidity.

Our study has strengths because it targets randomly selected samples from the total population in South Korea. This investigation of the prevalence of PI among the total population will help policy makers establish effective health care policies against the increasing medical costs. Recent studies have evaluated the prevalence of PI among hospitalized patients. In the United States, the International Pressure Ulcer Prevalence ™ Survey, facilitated by Hill-Rom, Inc. (Batesville, IN, USA), has been conducted since 1989 to assess the number and severity of pressure injuries in healthcare facilities [6], with the 2015 survey reporting the overall prevalence of PI to be 9.3%. In Europe, the European Pressure Ulcer Advisory Panel (EPUAP) introduced a reliable methodology to perform PI prevalence studies [22]. In a 2004 study, the PI prevalence was found to be 8.0% among 8515 hospitalized patients in Germany, 18.1% among 10,237 hospitalized patients in the Netherlands [7,8] and 8.9% among 37,307 patients in France [9]. However, the cohorts in these studies were not representative of the entire population of each country, and included only patients admitted to hospitals that voluntarily participated in the study. So the prevalence rates reported in these studies might be somewhat higher than the current study.

Our study showed a steady increase in the prevalence of PIs from 0.74% in 2009 to 0.86% in 2015. This may be due to an aging population, based on the finding that the standardized number of PIs was much higher among the elderly. South Korea is the fastest aging country among the Organization for Economic Co-operation and Development (OECD) countries, where individuals over the age of 65 accounted for 12.8% of the total population in 2015 [23]. Both the characteristics of PI that usually involve the elderly and the progress towards an aging society would have contributed to the increase in its overall prevalence. Moreover, the poverty rate among the elderly is the highest in Korea among all the OECD countries. Therefore, the relative cost burden of managing PIs in the elderly will be even higher than the younger population [24]. 

While numerous studies have revealed that chronic diseases such as hypertension, diabetes, and chronic obstructive pulmonary disease are associated with low SES [14,15,16,17], only few studies have examined the relationship between PIs and low SES. There was a retrospective analysis of the development of PIs in spinal cord injured patients in Spain, which showed that the severity of PIs was higher in less educated subjects, though the finding was not significant [25]. In 2012, there was a cross sectional study on the relationship between PIs and health care access in spinal cord injured patients in the United States [26]. However, these two studies have a limitation in that they are based on a specific disease group such as spinal cord injured patients. 

On the contrary, our study evaluated a representative sample of PI patients from the whole country and classified them based on the type of national insurance which is an objective database. Our findings revealed a positive relationship between PI and SES. There can be several reasons for the high prevalence of PIs in people with low SES. In our study, MA beneficiaries had a lower rate of visits to tertiary teaching hospitals where high quality PI management is provided compared to NHI beneficiaries. They also had a higher proportion of patients visiting non-metropolitan area hospitals where medical accessibility is poor. These results indicate that the lower the SES, the lower is the quality of medical care one receives. Moreover, the less educated people are also more likely to have a lower interest in their health and therefore, will not visit hospitals for mild PIs [27]. In addition, chronic diseases with a higher prevalence in patients with low SES are accompanied by complications such as immobility, and sensory or vascular disorders, which are risk factors for PIs [14,15,16,17,28]. Therefore, PIs should be recognized as one of the diseases heavily influenced by health inequality. There have been many national initiatives in the United States to reduce PIs. In 2006, the Joint Commission included ‘Prevent health care-associated pressure ulcers’ as the 14th National Patient Safety Goal [29]. In 2007, the Centers for Medicare & Medicaid Services announced that acute care payments would be discontinued on 1 October 2008, for the ancillary care of hospital-acquired PIs [30]. The American Nurses Association has included “prevention of facility acquired pressure ulcer” as one of the items in the nursing quality evaluation in an attempt to prevent PIs [31]. In contrast, although the disease burden of PIs is high enough to be comparable to that of other severe diseases in Korea, there is a lack of social interest in PIs, and therefore, its management is limited to the area of nursing care. Active and systematic intervention of health care professionals is needed to reduce the prevalence of PIs.

Although there have been many studies on the risk factors for PI in various settings, there have been only a few on the comorbidities associated with PIs. In our study, the average CCI of patients with PIs was over 2.0, which can be interpreted as 1-year mortality exceeding 20% [21]. This is a remarkably high figure, compared to the 5-year mortalities of four major cancers (thyroid, stomach, colon, and breast cancer) which are between 0% and 26% in Korea [32]. Also, the results of logistic regression analysis indicate that high CCI is associated with a high prevalence of PIs, suggesting that patients with more severe co-morbidities require more stringent care for PIs. The Korean NHI Corporation operates a special program for deducting out of pocket fees for patients with cancer, rare incurable diseases, heart disease, cerebrovascular disease, severe burns, and serious trauma. Based on our findings, it is necessary to reduce the financial burden of patients with PIs too, considering its comorbidities, high mortalities, and high prevalence in people with low SES. 

In the study on special comorbidities, we found that more than 10% of the PI inpatients also had plegia, diabetes, and sepsis. We observed more PIs in patients with spastic paralysis compared to those with flaccid paralysis, which is contrary to some previous studies that have reported PIs to be more common in patients with flaccid paralysis because of muscle wasting and decreased tissue viability [33]. Our findings may be explained by the fact that severe spasticity will lead to joint contractures, friction, shear, and mobility impairment. Besides, the shearing movements caused by the spastic response may result in skin breakdown [34]. Therefore, in patients with spastic paralysis, spasticity control should also be considered when treating the PIs in these patients.

There are some limitations to our study. First, although the statistical surveys based on the HIRA database have advantages in terms of representativeness, objectivity and cost effectiveness, there is a possibility that the status of the patient is not accurately reflected in the diagnostic code [35]. This could be a simple diagnostic error or an inability to meet the insurance coverage criteria, but it is likely that diagnostic codes of diseases that are easily overlooked, such as PIs, are not registered if they are mild. Second, we classified the SES of patients only based on the type of health insurance they had. We will, therefore, be further researching the SES based on socioeconomic factors categorized by income level, education level, and type of occupation. Third, we wanted to gather information on the stage and location of the PIs according to ICD-10th codes. Unfortunately, most electronic medical record programs used in Korea are not capable of registering specific diagnostic codes. So, we are going to report this issue to the Korean government and the HIRA, and we are going to carry out another study using electronic medical records from each individual hospital. Forth, we investigated the proportion of comorbidities only in the PI inpatients without considering the proportion of comorbidity among all patients. Therefore, a cohort study considering a causal relationship with clinically associated diseases should be conducted to be a more meaningful comorbidity study. Lastly, we were not able to estimate the incidence rate of PI because of the inherent limitations to the NIS database. Besides, our data may not reflect PI patients who live in the community and are not inpatients. Therefore, further study is needed to gain more accurate epidemiological data.

## 5. Conclusions

This study has for the first time revealed the increasing prevalence of PIs among the total population of South Korea. Our results indicate that the medical cost for treatment and management of PIs will increase in the future, and it will be accelerated due to the rapidly aging society. In addition, low SES and severe comorbidities are associated with a high prevalence of PIs. The occurrence of PIs will be relatively more burdensome to these sections of the population, threatening their household economy and further reducing the quality of life. Therefore, PIs should not be overlooked as the responsibility of just the nursing care professionals, but should be recognized as one of the serious diseases. The establishment of an intense medical care system is needed to not only to reduce the prevalence of PIs but also to increase the awareness in people with low SES.

## Figures and Tables

**Table 1:** Prevalence of PIs from 2009 to 2015 in South Korea.

Category		Year, n (%)						
		2009	2010	2011	2012	2013	2014	2015
Study population	Total	1,116,040 (100.0)	1,136,688 (100.0)	1,165,113 (100.0)	1,169,428 (100.0)	1,163,117 (100.0)	1,269,210 (100.0)	1,293,144 (100.0)
	Male	517,123 (46.3)	522,237 (45.9)	535,030 (45.9)	535,455 (45.8)	535,045 (46.0)	588,179 (46.3)	599,093 (46.3)
	Female	598,917 (53.7)	614,451 (54.1)	630,083 (54.1)	633,973 (54.2)	628,072 (54.0)	681,031 (53.7)	694,051 (53.7)
	Mean age (SD)	41.5 (22.5)	42.8 (22.4)	43.5 (22.3)	44.6 (21.8)	45.7 (21.4)	43.9 (23.0)	44.1 (23.2)
N. of PI inpatients	Total	12,375 (1.1)	13,681 (1.2)	13,892 (1.2)	14,187 (1.2)	14,765 (1.3)	15,373 (1.2)	16,202 (1.3)
	Male	5963 (0.5)	6788 (0.6)	6805 (0.6)	6993 (0.6)	7183 (0.6)	7421 (0.6)	7924 (0.6)
	Female	6412 (0.6)	6893 (0.6)	7087 (0.6)	7194 (0.6)	7582 (0.7)	7952 (0.6)	8278 (0.6)
	Mean age (SD)	71.4 (14.8)	71.6 (16.7)	71.9 (16.9)	72.4 (14.6)	72.5 (14.8)	72.9 (15.1)	73.1 (15.1)
Standardized N. of annual PI inpatient^1^		736	797	789	785	816	831	861
N. of PI inpatients in South Korea^2^		95,192	105,238	106,861	109,129	113,576	118,253	124,629

PI: pressure injury; SD: standard deviation. ^1^ Annual prevalence of pressure injury was standardized number per population size 100,000 using 2015 census data surveyed by Statistics Korea. ^2^ N. of PI inpatients relative to the total population in South Korea was calculated by applying sampling weight.

**Table 2:** Number of PI inpatients by the type of health insurance, regional location and level of medical institution.

	Category	Year, n (%)						
		2009	2010	2011	2012	2013	2014	2015
N. of PI inpatients		12,375 (100.0)	13,681 (100.0)	13,892 (100.0)	14,187 (100.0 )	14,765 (100.0)	15,373 (100.0)	16,202 (100.0)
Type of health insurance	NHI	9650 (78.0)	10,795 (78.9)	10,966 (78.9)	11,283 (79.5)	11,950 (80.9)	12,484 (81.2)	13,285 (82.0)
	MA	2725 (28.2)	2886 (26.7)	2926 (26.7)	2904 (25.7)	2815 (23.6)	2889 (23.1)	2917 (22.0)
Region of medical institution	METP		6189 (45.2)	6285 (45.2)	6408 (45.2)	6721 (45.5)	7095 (46.2)	7522 (46.4)
	Non-METP		7492 (54.8)	7607 (54.8)	7779 (54.8)	8044 (54.5)	8278 (53.8)	8680 (53.6)
NHI	METP		5009 (46.4)	5048 (46.1)	5197 (46.1)	5521 (46.2)	5812 (46.6)	6228 (46.9)
	Non-METP		5786 (53.6)	5918 (54.0)	6086 (53.9)	6429 (53.8)	6672 (53.5)	7057 (53.2)
MA	METP		1180 (40.9)	1237 (42.3)	1211 (41.7)	1200 (42.6)	1283 (44.4)	1294 (44.4)
	Non-METP		1706 (59.1)	1689 (57.8)	1693 (58.3)	1615 (57.4)	1606 (55.6)	1623 (55.7)
Level of medical institution	TTH	1975 (16.0)	2347 (17.2)	2300 (16.6)	1612 (11.4)	1750 (11.9)	2709 (17.6)	3174 (19.6)
	GH	3968 (32.1)	4523 (33.1)	4726 (34.0)	5510 (38.8)	5667 (38.4)	5005 (32.6)	5042 (31.1)
	H	5687 (46.0)	5987 (43.8)	5971 (43.0)	6170 (43.5)	6428 (43.5)	6678 (43.5)	7008 (43.3)
	C	745 (6.0)	820 (6.0)	886 (6.4)	895 (6.3)	920 (6.2)	976 (6.4)	969 (6.0)
NHI	TTH	1773 (18.4)	2104 (19.5)	2057 (18.8)	1450 (12.9)	1585 (13.3)	2441 (19.6)	2823 (21.3)
	GH	3054 (31.6)	3567 (33.1)	3691 (33.7)	4412 (39.1)	4602 (38.5)	3986 (31.9)	4106 (30.9)
	H	4227 (43.8)	4485 (41.6)	4518 (41.2)	4703 (41.7)	4983 (41.7)	5239 (42.0)	5549 (41.8)
	C	596 (6.2)	636 (5.9)	694 (6.3)	718 (6.4)	780 (6.5)	814 (6.5)	799 (6.0)
MA	TTH	202 (7.4)	243 (8.4)	243 (8.3)	162 (5.6)	165 (5.9)	268 (9.3)	351 (12.0)
	GH	914 (33.5)	956 (33.1)	1035 (35.4)	1098 (37.8)	1065 (37.8)	1019 (35.3)	936 (32.1)
	H	1460 (53.6)	1502 (52.1)	1453 (49.7)	1467 (50.5)	1445 (51.3)	1439 (49.8)	1459 (50.0)
	C	149 (5.5)	184 (6.4)	192 (6.6)	177 (6.1)	140 (5.0)	162 (5.6)	170 (5.8)

PI: pressure injury, NHI: National Health Insurance, MA: Medical Aid, METP: Metropolitan, Non-METP: Non-metropolitan, TTH: Tertiary teaching hospital, GH: General hospital, H: Hospital, C; Clinic. ^1^ In the years of 2010, 2011, 2013, and 2015, there are differences between total and sub groups because of no information on the level of medical institutions. ^2^ Clinics include private, public and others.

**Table 3:** Average CCI of PI inpatients and the number of PI inpatient with special comorbidities.

Variable		Year, n (%)						
		2009	2010	2011	2012	2013	2014	2015
N. of PI inpatient		12,375 (100.0)	13,681 (100.0)	13,892 (100.0)	14,187 (100.0)	14,765 (100.0)	15,373 (100.0)	16,202 (100.0)
Average CCI [STD]		2.3 [2.1]	2.2 [2.0]	2.0 [1.9]	2.2 [2.0]	2.1 [1.9]	2.2 [2.0]	2.1 [1.9]
N. of PI inpatient with special comorbidities								
Plegia		2141 (17.3)	2313 (16.9)	2137 (15.4)	2037 (14.4)	2069 (14.0)	2169 (14.1)	2203 (13.6)
	Quadriplegia	508 (4.1)	554 (4.0)	542 (3.9)	543 (3.8)	581 (3.9)	657 (4.3)	634 (3.9)
	Hemiplegia	1136 (9.2)	1282 (9.4)	1013 (7.3)	932 (6.6)	909 (6.2)	937 (6.1)	934 (5.8)
	Monoplegia	61 (0.5)	59 (0.4)	77 (0.6)	46 (0.3)	52 (0.4)	44 (0.3)	58 (0.4)
	Paraplegia	169 (1.4)	218 (1.6)	227 (1.6)	226 (1.6)	214 (1.4)	257 (1.7)	246 (1.5)
	Flaccid paralysis	187 (1.5)	181 (1.3)	211 (1.5)	195 (1.4)	218 (1.5)	214 (1.4)	240 (1.5)
	Spastic paralysis	580 (4.7)	643 (4.7)	563 (4.1)	543 (3.8)	510 (3.5)	522 (3.4)	489 (3.0)
Diabetes		1275 (10.3)	1432 (10.5)	1586 (11.4)	1697 (12.0)	1706 (11.6)	1917 (12.5)	2055 (12.7)
Sepsis		1467 (11.9)	1596 (11.7)	1531 (11.0)	1637 (11.5)	1787 (12.1)	1892 (12.3)	1960 (12.1)

PI: pressure injury, CCI: Charlson Comorbidity Index, STD: standardized deviation.

**Table 4:** Logistic regression analysis between PI diagnosis and socio-demographic factors among the overall population that used medical services between 2009 and 2015 in Korea.

Variable	Category	Year, Odds Ratio (95% Confidence Interval)			
		2009	2011	2013	2015
Total study population		1,116,040	1,165,113	1,163,117	1,293,144
N. of PI inpatients		12,375	13,892	14,765	16,202
Sex	Male	1.00	1.00	1.00	1.00
	Female	0.84 (0.82–0.85)	0.84 (0.83–0.86)	0.85 (0.83–0.86)	0.85 (0.83–0.86)
Age	0–39	1.00	1.00	1.00	1.00
	40–64	0.66 (0.64–0.69)	0.66 (0.64–0.69)	0.65 (0.62–0.67)	0.65 (0.63–0.67)
	65–74	1.98 (1.91–2.06)	1.90 (1.83–1.97)	1.79 (1.72–1.86)	1.78 (1.72–1.84)
	75+	5.58 (5.39–5.77)	5.29 (5.12–5.47)	5.15 (4.98–5.32)	5.29 (5.13–5.46)
Type of health insurance	NHI	1.00	1.00	1.00	1.00
	MA	1.45 (1.41–1.48)	1.49 (1.46–1.52)	1.45 (1.42–1.49)	1.46 (1.43–1.49)
CCI	0	1.00	1.00	1.00	1.00
	1-2	1.11 (1.08–1.14)	1.05 (1.03–1.08)	1.13 (1.10–1.15)	1.10 (1.07–1.12)
	3+	1.96 (1.91–2.02)	2.07 (2.02–2.13)	1.99 (1.94–2.05)	1.99 (1.94–2.04)
Plegia comorbidity	NO	1.00	1.00	1.00	1.00
	YES	2.48 (2.39–2.58)	2.17 (2.09–2.26)	2.05 (1.98–2.13)	1.88 (1.81–1.95)

PI: pressure injury, NHI: National Health Insurance, MA: Medical Aid, CCI: Charlson comorbidity index.
